# *Aggregatibacter actinomycetemcomitans* Leukotoxin (LtxA; Leukothera^®^): Mechanisms of Action and Therapeutic Applications

**DOI:** 10.3390/toxins11090489

**Published:** 2019-08-26

**Authors:** Brian A. Vega, Benjamin A. Belinka Jr., Scott C. Kachlany

**Affiliations:** 1 Department of Oral Biology, Rutgers School of Dental Medicine, Newark, NJ 07103, USA; 2 Actinobac Biomed, Inc., Princeton, NJ 08540, USA

**Keywords:** leukotoxin (LtxA), RTX (repeats-in-toxin) toxin, *Aggregatibacter actinomycetemcomitans*, lymphocyte function-associated antigen-1 (LFA-1), β_2_ integrins, cell death, oral microbiology, virulence factor, toxin therapy

## Abstract

*Aggregatibacter actinomycetemcomitans* is an oral pathogen that produces the RTX toxin, leukotoxin (LtxA; Leukothera^®^). *A. actinomycetemcomitans* is strongly associated with the development of localized aggressive periodontitis. LtxA acts as a virulence factor for *A. actinomycetemcomitans* to subvert the host immune response by binding to the β_2_ integrin lymphocyte function-associated antigen-1 (LFA-1; CD11a/CD18) on white blood cells (WBCs), causing cell death. In this paper, we reviewed the state of knowledge on LtxA interaction with WBCs and the subsequent mechanisms of induced cell death. Finally, we touched on the potential therapeutic applications of LtxA (trade name Leukothera^®^) toxin therapy for the treatment of hematological malignancies and immune-mediated diseases.

## 1. Introduction

*Aggregatibacter actinomycetemcomitans* is a Gram-negative oral pathogen that is strongly associated with the development of localized aggressive periodontitis [1,2]. *A. actinomycetemcomitans* belongs to the HACEK group of bacteria that can cause systemic diseases, including endocarditis [3]. 

In order to colonize, cause disease, and persist in the host, *A. actinomycetemcomitans* produces an arsenal of virulence factors. These virulence factors include adherence factors, biofilm polysaccharides, lipopolysaccharides (LPS), and toxins [1]. *A. actinomycetemcomitans* produces two important protein toxins—cytolethal distending toxin (CDT) [4,5] and leukotoxin (LtxA) [6,7]. Both CDT and LtxA play important roles in *A. actinomycetemcomitans* subversion of the host immune response, but differ in target cell specificity and expression pattern. This review focused on LtxA. 

*A. actinomycetemcomitans* LtxA is a secreted protein of 114 kDa [8]. The amount of LtxA secreted by *A. actinomycetemcomitans* depends on the size of the leukotoxin promoter region [9]. Minimally leukotoxic 652 strains contain the full-length, 1000 base-pair (bp) promoter, while the highly leukotoxic JP2 strains harbor a 532-bp deletion at the 3’ end of the promoter [9]. It is believed that this 532-bp deletion removes a transcriptional terminator of *ltxA* expression [10]. The *ltx* operon (*ltxCABD*) encodes four genes. *ltxC* is the first gene in the operon and is required for the post-translational acylation of internal lysine residues necessary for toxin activity [11,12,13]. *ltxA* is the second gene in the operon and encodes the full-length toxin. The production of LtxA is regulated by a number of factors, including the level of fermentable sugars, pH, and oxygen levels [14,15,16,17]. *ltxB* and *ltxD* are the third and fourth genes in the operon and are required for toxin translocation and secretion [13,18,19,20,21]. LtxB localizes to the inner bacterial membrane and interacts with LtxD, which is on the periplasmic side of the inner membrane. LtxB and LtxD interact with a third protein, TdeA (for Toxin and Drug Export) [18,22], on the outer bacterial membrane to form a type I secretion system for LtxA export. Further discussion, including schematic depictions, of the *ltx* operon [23], regulation of ltxA transcription [10], and regulation of LtxA production [23] can be found in previous publications.

LtxA is a member of the RTX (repeats-in-toxin) toxin family that includes *Escherichia coli* α-hemolysin (HlyA), *Bordetella pertussis* adenylate cyclase (CyaA), *Mannheimia haemolytica* leukotoxin (LktA), *Vibrio cholerae* Rtx toxin (RtxA), among other Gram-negative pathogens [6,7,24]. *A. actinomycetemcomitans* LtxA shares approximately 51% amino acid sequence similarity with *E. coli* HlyA and is 43% identical to *M. haemolytica* LktA. *E. coli* HlyA and *B. pertussis* CyaA intoxicate human leukocytes while *M. haemolytica* LktA intoxicates bovine leukocytes. Historically, RTX toxins are characterized by their similar structural features and are believed to interact with the plasma membrane of target cells in analogous manners. At the N-terminus, there are conserved amphipathic helices believed to be involved in toxin interaction with host cell membrane and receptor [13,25,26,27]. In the center of the protein are two lysine residues that are covalently modified with fatty acid residues [11,12,28,29]. The repeats domain encompasses the characteristic nonapeptide glycine-rich repeats, containing the consensus sequence GGXG(N/D)DX(L/I/F)X, which is responsible for binding calcium ions, a critical feature for maintaining cytotoxicity [25,30]. LtxA has twelve such repeats [24]. At the C-terminus is a ~100-amino acid domain involved in secretion of the toxin by a type I secretion system. The structural domains of LtxA have been extensively characterized in other studies [31,32]. 

In this review, we focused on the biology of LtxA interaction with host cell membranes and receptors, the mechanisms by which LtxA intoxicates various subsets of white blood cells and the potential therapeutic applications of LtxA toxin therapy.

## 2. Interaction of LtxA with White Blood Cells

LtxA has long been known to have a very narrow host range specific for white blood cells (WBCs) of human and Old World primate origin [33,34,35], suggesting that the toxin binds to a specific cell surface receptor. To determine the regions of LtxA responsible for this species and cell type specificity, Lally et al. developed a chimeric toxin and determined that amino acid residues 688–941 are necessary for LtxA to kill target human cells [13]. This amino acid region contains the nonapeptide glycine-rich repeats, as well as 34 residues before and 95 residues after the repeats [13], providing further evidence that this repeated domain is critical for cytotoxicity [25,30]. LtxA contains 12 such repeats [24]. Chimeric LtxA containing only 9 of these repeat regions fails to kill target cells. Thus, these 12 nonapeptide glycine-rich repeat domains are essential for the unique species recognition of LtxA. 

### 2.1. Receptor Independent Interactions with Target Cell Membranes

Prior to interaction with its receptor, lymphocyte function-associated antigen-1 (LFA-1), LtxA may associate with the host cell plasma membrane and induce changes. The earliest observable effects of LtxA on target cells is an increase in cytosolic (Ca^2+^), followed by a decrease in membrane potential [36]. LtxA can adsorb to cell membranes of toxin-sensitive and toxin-resistant cells [37], but fails to perturb the cell membrane in toxin-resistant cells [36]. LtxA induced Ca^2+^ mobilization in toxin-sensitive and toxin-resistant cells, suggesting that this occurs in the absence of LFA-1 and is not sufficient for cell lysis [38]. Interestingly, when *ltxC* is deleted from the *ltx* operon, there is no increase in cytosolic (Ca^2+^) in response to LtxA [11,12]. Studies have proposed that initial adsorption of LtxA to the cell membrane occurs via insertion of these fatty acyl chains into the membrane of target cells [12,39]. This supports the model that LtxA-mediated cytotoxicity occurs in two phases and that the fatty acylations on LtxA are required for this initial phase of interaction of membrane interaction [39]. 

LtxA was believed to be a membrane damaging toxin, and initial morphological studies suggested that LtxA bent target cell membranes and formed pores that disrupted the osmotic equilibrium of the cell [40,41]. However, more recent studies found that while LtxA clustered on target cell surfaces, there were no breaks in the membrane that were consistent with pore formation [40]. LtxA-treated cells demonstrated two membrane defects—the collapse of the microvilli normally present on the outer surface of cells, and the formation of cell surface depressions, followed by lipid-lined cavities [40]. However, the membranes appeared to be continuous, suggesting that LtxA membrane disruptions are produced not by pore formation, but rather by membrane destabilization related to nonlamellar phase formation in a manner independent of LFA-1 [40]. Divalent cations, such as Ca^2+^, can promote nonlamellar phases [42,43], and interestingly, LtxA elevates cytosolic (Ca^2+^) in an LFA-1 independent manner [38]. Thus, LtxA-induced Ca^2+^ mobilization may aid in promoting nonlamellar phase formation and membrane destabilization. 

LtxA association with the lipid bilayer results in a structural change in LtxA that generates more α-helical content [44,45]. These distinct structural requirements for LtxA membrane binding and membrane destabilization suggest that they are independent events. This supports the proposed model that the hydrophobic residue-rich portion of LtxA inserts into the membrane, initiating formation of a nonlamellar structure, thus weakening and destabilizing the membrane [32,40,44,45]. Furthermore, LtxA has been shown to associate with the membrane in a conformation where the hydrophobic and C-terminal domains reside outside the cell membrane and the central and repeats domains are inside the membrane. Since the hydrophobic residues were found to reside externally to the membrane, they are not believed to be involved in membrane interactions. Interestingly, the fatty acylations on LtxA were found to be in the liposome, suggesting that these modifications may play a role in membrane interactions and cell association [32], providing a potential function for the post-translational modification of LtxA and perhaps other RTX toxins.

**Figure 1:**
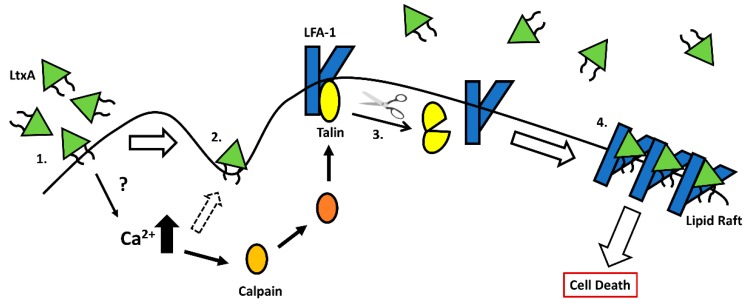
Model for LtxA association with host cell membranes. 1. LtxA (shown with fatty acyl chains) passively adsorbs onto cell membranes and anchors via the fatty acylation. This is believed to elevate intracellular calcium levels. 2. Association of LtxA with the target cell membranes results in lipid bilayer destabilization, demonstrated by the formation of cell surface depression. 3. The elevated calcium levels activate the calcium-dependent protease calpain. Calpain cleaves talin, a cytoskeletal tethering protein that holds LFA-1 in place in the cell membrane. 4. LFA-1 can cluster in the lipid raft compartment where LtxA interaction with LFA-1 is mediated by the fatty acyl chains at the membrane interface, allowing for the toxin to become partially embedded into the cell membrane. Downstream signaling results in cell death.

In addition to lipid bilayer destabilization, LtxA is known to almost irreversibly bind to cholesterol [31,38]. LtxA has a strong affinity for cholesterol and contains two cholesterol recognition/amino acid consensus (CRAC) sites—CRAC^336^ (^333^LEEYSKR^339^), which is highly conserved among RTX toxins, and CRAC^503^ (^501^VDYLK^505^), which is unique to LtxA [31]. Both CRAC sites are located within the N-terminal hydrophobic domain of LtxA. Only CRAC^336^ competitively inhibits LtxA binding and is essential for LtxA cytotoxicity [31,46,47]. The CRAC motifs on LtxA were found both inside and outside the plasma membrane, suggesting that these CRAC motifs are located near the LtxA membrane binding interface [32]. LtxA requires cholesterol for cytotoxicity [31,38]. The lipid raft domain is cholesterol rich and LtxA is proposed to bind cholesterol and aid in the localization and clustering of LFA-1 and LtxA in the lipid raft compartment—a key step in LtxA-mediated cell death [31,38]. LtxA has also been found to bound to cholesterol in non-raft membrane compartments and this interaction may be the cause of the LFA-1-independent increase in intracellular Ca^2+^ levels [38]. Following this increase in intracellular Ca^2+^ levels, the calcium d-pendent protease calpain becomes activated [38,48]. Calpain cleaves the cytoskeletal tethering protein, talin, which frees LFA-1, allowing for integrin clustering in the lipid rafts [38,49]. In the cholesterol-rich lipid raft, LtxA then binds to LFA-1 [38]. Figure 1 depicts a proposed model of LtxA interaction with target cell membranes and LFA-1. The nature of the LtxA/LFA-1 interaction is discussed below.

### 2.2. Interaction of LtxA with β_2_ Intergrins 

Lally et al. utilized a series of elegant studies to identify lymphocyte function-associated antigen-1 (LFA-1) as the receptor for LtxA [50]. Upon generation of a monoclonal antibody that inhibited LtxA-mediated cytolysis, affinity column purification pulled out two proteins that had complete homology to CD11a and CD18, the two protein chains that heterodimerize to comprise LFA-1. Passive adsorption of LtxA onto polystyrene beads allowed for co-immunoprecipitation of CD11a and CD18. Additionally, transfection of an expression plasmid containing the genes for CD11a and CD18 into toxin-resistant K562 cells conferred LtxA susceptibility to these cells. More recently, another study reported that LtxA can also bind and utilize Mac-1 (CD11b/CD18) and CR4 (CD11c/CD18) to intoxicate cells with comparable efficiencies [51]. 

LFA-1 is expressed on all white blood cells while Mac-1 and CR4 are only expressed on cells of myeloid origin and are regulated in their surface expression, unlike LFA-1 [52,53]. The alignment of amino acids sequences of CD11a, CD11b and CD11c reveals sequence identities of 34–35%, suggesting that LtxA may interact with these proteins similarly. While Mac-1 and CR4 have been shown to confer susceptibility to LtxA [51], more studies have characterized LtxA interactions with LFA-1 and thus, the remainder of this discussion will focus on that interaction.

LFA-1 is a key adhesion molecule that aids in leukocyte migration via interaction with the intercellular adhesion molecules (ICAMs) expressed on vascular endothelial cells [54]. LFA-1 exists in three conformational states—a resting state, an intermediate/extended state and an activated state. Resting state LFA-1 is unable to bind ICAM-1. Upon leukocyte activation, LFA-1 changes conformations to the intermediate/extended state which can weakly bind to ICAM-1, allowing for downstream signaling to initiate the full extension to the high affinity, activated form of LFA-1 that allows for tight adhesion and WBC migration to peripheral tissues [55,56]. Typically, interaction of integrins with their ligands results in enhanced survival, differentiation and other immunological events [57,58]. Thus, it is intriguing that LtxA interaction with LFA-1 stimulates cell death to eliminate the most immunologically relevant cells targeting *A. actinomycetemcomitans*. 

Previous studies have sought to characterize the nature of LtxA interaction with LFA-1. It appears that both CD11a and CD18 are required for LtxA to intoxicate cells. Chimeric receptor studies demonstrated that CD18 is the functional receptor for LtxA and confers species-specificity to LtxA [59]. Human cells expressing bovine CD11a/human CD18 were susceptible to LtxA, while human cells expressing human CD11a/bovine CD18 were resistant to the toxin. Furthermore, a 100-amino acid residue region of the cysteine-rich tandem repeats encompassing integrin-epidermal growth-factor-like domains 2, 3, and 4 of the extracellular domain of CD18 are critical for toxin activity against LFA-1 expressing cells [59]. Another study showed that an N-terminal 128 amino acid domain encompassing the β sheets 1 and 2 of the β-propeller domain of CD11a is important for recognition by LtxA [60]. These studies suggest that LtxA comes in contact with both CD11a and CD18; however, it has been hypothesized that CD18 harbors the major toxin binding site due to the ability of LtxA to utilize Mac-1 and CR4 [51]. Studies have also demonstrated that LtxA recognizes CD11a/CD18 through N-linked oligosaccharides on LFA-1, specifically via sialic acid residues, and this glycosylation is required for cytotoxic activity [61,62].

Two recent studies have proposed interesting interactions of LtxA with LFA-1. One proposed that LtxA binds to the extracellular domains of LFA-1 initiating receptor clustering in the lipid raft compartment, allowing the toxin to transverse the membrane where it binds the cytoplasmic domains of CD11a and CD18 with a strong affinity, suggesting that LtxA may exist partially embedded in the cell membrane [63]. It is believed that LtxA membrane association via CRAC motifs allows LtxA to adopt a conformation amenable to membrane insertion and association with both extra- and intra-cellular domains of CD11a and CD18 [32]. Another study proposed that LtxA does not require the cytoplasmic domain of CD18, suggesting downstream integrin signaling is not required for cytotoxicity [64]. Further work is required to resolve the interaction of LtxA with LFA-1 and other β_2_ integrins.

## 3. Mechanisms of Action

The sensitivity of WBCs to LtxA is directly correlated to the level of LFA-1 surface expression [35]. Cells which expressed the highest levels of LFA-1 on the cell surface were the most sensitive to LtxA. LtxA preferentially targets the WBCs expressing the active conformation of LFA-1 [65,66]. Thus, LtxA targets the most immunologically relevant cells, allowing *A. actinomycetemcomitans* to persist, and under the appropriate conditions, causes disease. At high doses, LtxA is believed to destroy cells via pore formation in the cell membrane, a characteristic of RTX toxins, causing necrosis, while at lower doses of LtxA, apoptotic-like mechanisms of cell death eliminate cells [67,68,69]. The mechanisms of LtxA-mediated cell death, especially the events downstream of LFA-1, were poorly understood until recently. The current knowledge of the mechanisms by which LtxA kills various immune cell subsets is described below.

### 3.1. Monocytes and Macrophages

Initially it was unclear if LtxA initiated apoptosis or necrosis in immune cells in the monocytic lineage. Promyelocytic HL-60 cells treated with LtxA exhibited characteristic features of apoptotic cells, including reductions in cell size, organelle condensation, membrane budding, translocation of phosphatidylserine from the inner to the outer leaflet of the plasma membrane, and DNA fragmentation [67]. Among leukocytes, monocytes are the most sensitive to LtxA [70] and the kinetics of this process are very rapid [66].

LtxA has been shown to preferentially target CD14^+^ monocytes that express the purinergic receptor, P2X_7_R [71]. LtxA is known to increase cytosolic (Ca^2+^) through a receptor-independent mechanism [38] and interestingly, the inhibition of P2X_1_R prevents cytosolic (Ca^2+^) spikes, and P2X_7_R was found to participate in sustaining this (Ca^2+^) spike [72]. CD14^+^ P2X_7_R expressing cells respond to LtxA with a rapid release of ATP to the extracellular milieu that activates P2X receptors, allowing Ca^2+^ entry into the cell. LtxA-induced (Ca^2+^) spikes were at least partially secondary to P2XR activation [72], suggesting that P2XR activation may cause Ca^2+^ mobilization in response to LtxA. Thus, the activation of P2XR may precede LtxA interaction with LFA-1. Inhibition of P2X_1_R, P2X_4_R, and P2X_7_R prevented LtxA-mediated cell death of the CD14^+^ cell population, indicating an important role of P2X_7_R signaling in cell death [71,72]. It is unclear if P2X receptors are important in LtxA-mediated cell death of other immune cell subsets.

Initial studies on LtxA interaction with host cells demonstrated that LFA-1 was the cellular receptor [50]. More recently, studies identified that LtxA was able to kill K562 cells transfected to express Mac-1 (CD11b/CD18) and CR4 (CD11c/CD18) just as efficiently as those expressing LFA-1 (CD11a/CD18) [51]. In support of this, we have preliminary data showing that THP-1 monocytes with CRISPR/Cas9-mediated deletion of CD11a remain sensitive to LtxA, albeit at lower levels than their wild-type counterparts (Vega, BA and Kachlany, SC, unpublished data). LtxA interaction with LFA-1 lead to dephosphorylation and thus, activation of the ubiquitous actin-binding protein, cofilin, which lead to enhanced clustering of LFA-1 on the surface of THP-1 monocytes [73]. Following clustering of LFA-1 in the lipid raft compartment and LtxA binding to LFA-1, the LtxA/LFA-1 complex is internalized via a receptor-mediated endocytosis mechanism [66]. Because cofilin regulates actin dynamics, its activation in response to LtxA may result in increased LFA-1 trafficking and internalization of the LFA-1/LtxA complex. 

Once bound to LFA-1, LtxA treatment results in rapid phosphorylation of MAPK p38 and activation of caspase-1 [70,71]. The inhibition of caspase-1 was found to prevent LtxA-mediated cell death in monocytes, suggesting a critical role in the cell death cascade for this protease. The cleavage of caspase-1 and activation of the inflammasome is a precursor to bioprocessing and secretion IL-1β and IL-18, and abundant IL-1 β and IL-18 are produced and released in response to LtxA [70,74,75,76]. This caspase-1 dependent programmed cell death that leads to secretion of IL-1β and IL-18 closely resembles the pro-inflammatory cell death pathway of pyroptosis [77]. However, a separate study demonstrated that while LtxA induced dose-dependent caspase activation in THP-1 monocytes, the inhibition of caspases still led to potent cell death [66], suggesting that LtxA activates another, more prominent cell death pathway in THP-1 monocytes. 

**Figure 2:**
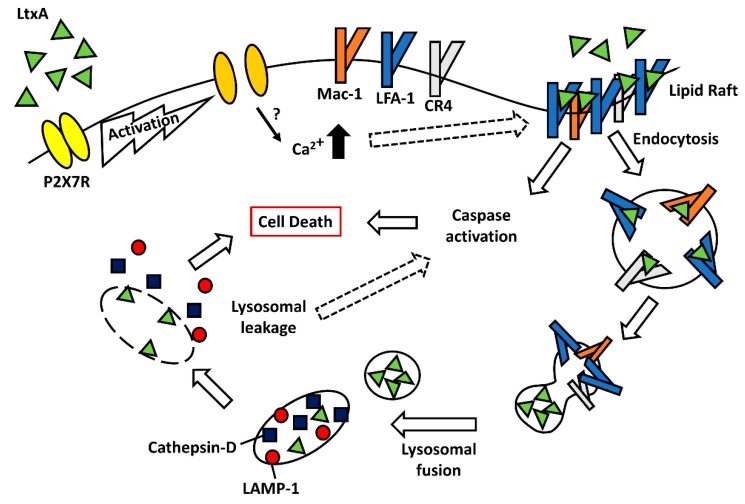
Model of LtxA-mediated cell death in monocytes. LtxA preferentially targets CD14^+^ monocytes expressing P2X_7_R. Upon treatment with LtxA, monocytes rapidly release ATP into the extracellular milieu, activating P2X_7_R. Activation of P2X_7_R allows Ca^2+^ to be mobilized, resulting in the clustering of LFA-1 and other β_2_ integrins into the lipid raft compartment. LtxA/LFA-1 interaction leads to the activation of caspases. LtxA is internalized in an LFA-1/ β_2_ integrin-dependent manner where LtxA is shuttled to the lysosome. LtxA ruptures the lysosomal membrane, allowing leakage of lysosomal proteases, such as Cathepsin D, into the cytosol leading to rapid and irreversible cell death.

The inhibition of numerous cell death pathways, including necrosis and necroptosis, failed to inhibit LtxA-mediated cytolysis in monocytes. However, pretreatment of cells with the phosphatidylinositol kinase (PI3K) inhibitor 3-MA, was able to significantly inhibit LtxA-mediated killing [66]. 3-MA has been used to inhibit lysosomal mediated cell death pathways, such as autophagy, which lead to investigation into the role of lysosomes in LtxA-mediated cell death in THP-1 cells. Confocal microscopy and flow cytometry revealed LtxA treatment leads to rapid disruption of the lysosomal membrane and acidification of the cytosol [66,78]. LtxA-treated cells exhibited membrane blebbing and altered cellular morphologies prior to lysosomal damage. This can occur in cellular degranulation, which has been proposed as a mechanism of LtxA-mediated cell death [79]. Degranulation requires the presentation of LAMP-1 (CD107a) on the surface of cells [80], which is not observed upon LtxA treatment [66]. The lysosomal aspartyl protease Cathepsin D was detected in cytosolic fractions of LtxA-treated cells and the inhibition of Cathepsin D with pepstatin A significantly inhibited killing of THP-1 cells. Furthermore, after LtxA is internalized into cells, LtxA is shuttled to the lysosomal membrane, where it causes its rupture and release of contents. It is proposed that release of these lysosomal proteases leads to the caspase activation observed. LtxA is the first bacterial toxin shown to localize to the lysosomal compartment where it causes the release of its contents into the cytosol. This lysosomal-mediated cell death pathway appears to occur in monocytes irrespective of which β_2_ integrin LtxA binds (Vega, BA and Kachlany, SC, unpublished data). Therefore, LtxA induces cell death in monocytes via disruption of lysosomes, causing a release of contents to the cytosol, resulting in its acidification in a manner distinct from degranulation. Figure 2 depicts a proposed model of LtxA-mediated cell death in monocytes and macrophages.

### 3.2. Polymorphonuclear Leukocytes

Neutrophils make up the largest population of polymorphonuclear leukocytes (PMNs) in the circulation, while eosinophils, basophils and mast cells are less abundant. As such, most of the mechanistic studies of the effects of LtxA on PMNs have been done primarily in neutrophils. 

Electron micrographs of PMNs treated with LtxA revealed rapid morphological changes. Shortly after the addition of LtxA, PMNs demonstrated signs of granule translocation toward the cell surface, the formation and shedding of cell membrane blebs, and karyorrhexis [79,81]. PMNs rapidly release the granule components resistin [82], matrix metalloproteinase 8 [83], myeloperoxidase, lysozyme, β-glucuronidase [84,85], lactoferrin [79], neutrophil elastase [79,86] and other lysosomal enzymes into the extracellular compartment. The release of these granule components was accompanied by a decrease in the number of intracellular granules seen via electron microscopy [79]. Granule release occurred prior to cell lysis, as extracellular lactate dehydrogenase levels remained low. This was intriguing since LtxA lysis of PMNs is believed to occur through pore formation but allows no efflux of cytosolic macromolecules, which may suggest that LtxA does not form pores in PMNs. LtxA treatment increased the levels of CD63 and CD66b (from primary and secondary granules, respectively) on the surface of the cell [79]. Conflicting reports on the involvement of LFA-1 in LtxA-induced PMN degranulation have been published [79,82].

Interestingly, several studies proposed that neutrophils exposed to LtxA can activate and release neutrophil extracellular traps (NETs) in a process called NETosis. NETosis is an innate defense strategy whereby neutrophils release DNA and certain granule components, such as neutrophil elastase, myeloperoxidase and lactoferrin [87,88], to the extracellular space where web-like DNA threads trap bacteria and bacterial products to thwart microbial infections. Neutrophils treated with LtxA quickly demonstrated signs of cell swelling. NET formation was observed after a 3-h incubation with LtxA, suggesting that cell swelling is a slow process, which allows NETosis to proceed to cell death/lysis in a controlled, perhaps distinct process [89,90,91]. Because NETosis is known to cause the release of granule contents and occur prior to neutrophil lysis, it is possible that the degranulation which precedes LtxA-induced neutrophil lysis [79] is due to NETosis. While degranulation is believed to be LFA-1-independent, studies have previously shown LFA-1-dependent NET release [92], which means that degranulation and NETosis could be two separate cell death pathways. Therefore, LtxA interaction with LFA-1 on neutrophils may induce rapid lysis and degranulation in some cells and/or NETosis and slow cell death in others; however, further work is necessary to resolve the mechanism of LtxA intoxication of PMNs. It is important to note that the lack of immortalized neutrophil cell lines and their short in vivo half-life pose a challenge to studying LtxA-mediated cell death in PMNs.

### 3.3. Lymphocytes

Lymphocytes were originally considered resistant to LtxA-mediated cytotoxicity [85,93]. Exposure of lymphocytes to LtxA resulted in very few cells, showing evidence of cell death using viability stains; however, electron microscopy and flow cytometry revealed cellular alterations indicative of apoptosis [69]. LtxA-induced cell death in lymphocytes occurs much slower than cell death in cells of myeloid origin.

Initial studies identified that upon binding to LFA-1 on lymphocytes, LtxA is not internalized via receptor-mediated endocytosis nor does LtxA cause disruption of the lysosomes [66]. However, a more recent study showed that while LtxA associates with host cell membranes independent of LFA-1, LtxA is at least partially internalized in Jn.9 cells in an LFA-1 dependent manner [63]. Using flow cytometry and fluorescence microscopy, LtxA was found to translocate into the cytosol and remained very close to the plasma membrane. The proximity of LtxA to the cell membrane may explain why previous studies did not observe toxin internalization in lymphocytes. LtxA has strong binding affinities for the membrane proximal region of CD11a and the intermediate domain of CD18 [63]. When bound to these domains of CD11a and CD18, the toxin brings the cytoplasmic domains of CD11a and CD18 closer together, which interferes with LFA-1 activation [63]. Interestingly, LtxA binds CD11a in close proximity to the region that Rap1 GTPase interacts with CD11a. Rap1 GTPase is activated by cytosolic Ca^2+^ increases and diacylglycerol and binds its effector molecule, RapL, on specific residues within CD11a to stimulate inside-out integrin signaling [94,95]. Thus, LtxA interaction with the membrane proximal region of CD11a could block integrin inside-out signaling. Rap1 and RapL are known to be involved in parts of apoptotic signaling pathways [96,97], and LtxA blockage of canonical integrin signaling could alter signaling pathways, thus allowing cell death signaling to occur through the integrin.

Other studies have identified a critical role for the Fas (CD95) death receptor in LtxA-mediated cell death in T-lymphocytes [98,99]. Fas and CD11a were found to colocalize on the surface of Jurkat E6.1 cells more strongly than CD11a and CD18, the molecules that are known to heterodimerize as LFA-1 [98]. CRISPR/Cas9-mediated deletion of Fas prevented LtxA from intoxicating Jurkat E6.1 cells [99]. Additionally, use of Jurkat A3 cells, which are very sensitive to Fas mediated apoptosis, were more sensitive to LtxA than Jurkat E6.1 cells [98]. These results suggest that Fas plays a major role in LtxA-mediated cell death. While Fas was found to be an early event in LtxA-mediated cell death [99], the potential interplay between LtxA, LFA-1, and Fas has not been deciphered.

The proposal that LtxA does not require the cytoplasmic domain of CD18 [64] or that LtxA interferes with LFA-1 signaling [63] may help explain how LtxA can signal via the Fas-mediated cell death pathway in lymphocytes. It is noteworthy that Fas does not play a role in LtxA-mediated cell death in B-lymphocytes ([100] and Vega, BA and Kachlany, SC, unpublished data). Further work is required to decipher if LtxA utilizes another death receptor in B-lymphocytes.

Upon treatment of lymphocytes with LtxA, classical features of apoptosis, such as decreased cell size, increased cytoplasmic granularity and DNA fragmentation are observed [67]. Downstream of the receptor(s), LtxA kills lymphocytes by a caspase-dependent mechanism [98]. CRISPR/Cas9-mediated deletion of LFA-1 or Fas prevented the activation of caspases in response to LtxA [99]. While LtxA treatment induced cleavage of both caspase-8 and -9, only the inhibition of caspase-8 significantly prevented cell death [98]. This finding was further corroborated using caspase-8 mutant cells that were partially, yet significantly resistant to LtxA-mediated cytolysis. In canonical Fas-mediated apoptosis, the activation of caspase-8 can lead to type I extrinsic apoptosis where caspase-8 directly activates the executioner caspase-3, or type II extrinsic apoptosis where caspase-8 triggers a series of events leading to perturbation of the mitochondria [101]. LtxA treatment of JY B cells resulted in mitochondria distention where cristae were obliterated [101], a phenomenon that is consistent with apoptosis [102,103]. Furthermore, LtxA treatment induced a loss of mitochondrial membrane potential and hyperproduction of reactive oxygen intermediates [68,100]. Fas signaling played a critical role in LtxA-mediated mitochondrial membrane permeabilization in T-lymphocytes [99]. LtxA treatment led to a decrease in ATP levels [100]. Loss of mitochondrial membrane potential led to the translocation of cytochrome c into the cytoplasm and cleavage of caspases-9 and -3. LtxA-mediated caspase-3 activation triggered the terminal events in apoptosis, including cleavage of the DNA repair enzyme poly (ADP-ribose) polymerase (PARP) and DNA fragmentation. Overexpression of anti-apoptotic proteins Bcl-2 and Bcl-x_L_ prevented uncoupling of the electron transport chain from oxidative phosphorylation, cytochrome c release, effector caspase-3 and -7 activation, PARP cleavage, and DNA fragmentation [100].

**Figure 3:**
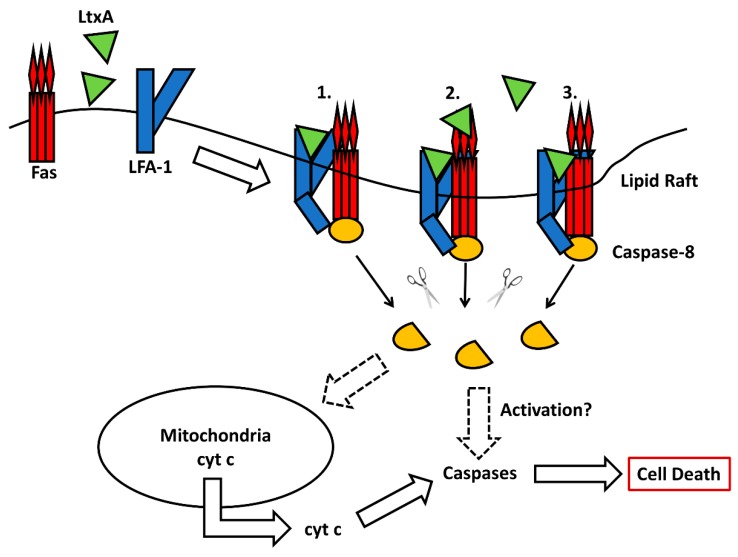
Model of LtxA-mediated cell death in lymphocytes. LtxA induces clustering of LFA-1 in the lipid rafts, where LFA-1 colocalizes strongly with Fas. After association with LFA-1, LtxA partially transverses the cell membrane and interacts with membrane proximal domains of LFA-1, interfering with its activation. The literature supports that LtxA only interacts with LFA-1 1, but it is possible that LtxA interacts with LFA-1 and Fas independently 2. or LtxA simultaneously interacts with both LFA-1 and Fas 3. Faulty activation of LFA-1 may allow the β_2_ tail to either directly activate caspase-8 or to signal via the Fas-mediated cell death pathway. The activation of caspase-8 can result in perturbation of the mitochondrial membrane, releasing cytochrome c into the cytosol, or further activation of effector caspases. Both pathways culminate in cell death.

These studies reveal many similarities between the mechanism of LtxA-mediated cell death in lymphocytes to that of canonical Fas-mediated apoptosis. Given the requirement of Fas for cell death [99], the colocalization of Fas and CD11a [98], and the similarities between the cell death signaling pathways, it may be plausible that LtxA interference of LFA-1 activation and signaling leads to apoptotic signaling via the canonical Fas-mediated cell death pathway in T-lymphocytes. These similarities warrant further investigation into the potential involvement of other death receptors in LtxA-mediated cell death in B-lymphocytes. A proposed model of LtxA-mediated cell death in lymphocytes is shown in Figure 3.

## 4. Potential Therapeutic Applications

Many bacterial toxins have been well studied because of the threats they pose to the host immune system. The natural targeting abilities of several bacterial toxins, such as those from *Clostridium botulinum* and *Corynebacterium diphtheriae* have been exploited by clinicians to treat diseases. *C. botulinum* neurotoxin (BOTOX) and *C. diphtheriae* diphtheria toxin (ONTAK) have been utilized clinically for the treatment of neuromuscular disorders [104] and T-cell lymphoma [105,106], respectively, among other toxin therapies.

LtxA is currently being investigated as a therapeutic agent (under tradename Leukothera^®^) for treatment of hematological malignancies and immune-mediated diseases. LFA-1 and other β_2_ integrins, the receptors for LtxA, are known to be overexpressed on WBCs in leukemias, lymphomas, autoimmune diseases and inflammatory disorders [107,108,109]. Additionally, LFA-1 is known to mediate migration of auto-reactive immune cells to various organs in autoimmune diseases, such as thyroiditis, psoriasis, multiple sclerosis, and rheumatoid arthritis [108]. Because LtxA specifically targets the active conformation of LFA-1 [65,66], LtxA may represent a novel targeted biotherapy for WBC disorders and is being developed as such. Several of the preclinical studies demonstrating the potential therapeutic efficacy of LtxA are described below. A summary of diseases in which β_2_ integrins play a role, and thus may be treatable with LtxA therapy, is provided in Table 1.

### 4.1. Leukemia and Lymphoma

Leukemia and lymphoma are cancers of WBCs that can be either acute or chronic diseases. These diseases are characterized by the uncontrolled proliferation of various subsets of immune cells. Many available standard chemotherapeutic agents are highly cytotoxic and have adverse side effects. LFA-1 is known to be overexpressed and over-activated in leukemias and lymphomas, leading to enhanced migration of diseased cells [107,109,110]. Due to the natural targeted potential of LtxA for these activated leukocytes [35,65,66], the therapeutic applicability of LtxA for the treatment of leukemia and lymphoma was evaluated in several studies.

LtxA has shown preclinical efficacy in SCID mouse xenograft models of human HL-60 myeloid leukemia and human RL B-cell lymphoma [35,98]. Treatment with LtxA resulted in complete tumor regression and enhanced overall survival. Peripheral blood mononuclear cells (PBMCs) from patients with acute monocytic leukemia were shown to be significantly more sensitive to LtxA than PBMCs from healthy controls [35]. Additionally, the expression levels of LFA-1 on the surface of cells correlated with the ability of LtxA to kill these cells—higher levels of LFA-1 lead to more killing by LtxA. Rhesus macaques injected intravenously with LtxA had transient decreases in WBCs post-infusion. LtxA had no effect on RBCs, platelets, hemoglobin, and other blood chemistry values and showed no signs of liver or kidney toxicity [35]. LtxA has also been shown to synergize in vitro with standard chemotherapeutic agents [134], which could allow for combination therapies that result in the administration of lower doses of more cytotoxic drugs, thus diminishing adverse effects. Due to the specificity of LtxA, it poses an advantage over chemotherapeutics because no off-target toxicities have been observed. Thus, LtxA may be an effective and safe novel therapeutic agent for the treatment of leukemia and lymphoma. LtxA is currently in pre-clinical development for the treatment of relapsed and refractory leukemia and lymphoma.

### 4.2. Psoriasis

Psoriasis is a common chronic skin disease that affects approximately 2–3% of the world’s population [135,136]. Psoriasis is an inflammatory skin disorder that is caused by over-reactive immune cells in the skin inducing rapid, abnormal proliferation and differentiation of epidermal keratinocytes that ultimately manifests on the skin surface as elevated, scaly lesions. Studies have shown that the WBCs involved in the progression of psoriasis have an upregulation and overactivation of LFA-1 [111,112]. While there is no cure for psoriasis, numerous therapeutic options for mild-to-moderate psoriasis, such as topical therapy and phototherapy, are available. Severe disease can be treated with methotrexate, retinoids, cyclosporine, and monoclonal antibodies, but there are known adverse effects [137,138]. LtxA is a much more targeted approach to treating psoriasis because it specifically depletes the immune cells involved in disease progression.

LtxA was shown to be highly effective at treating psoriasis in a human xenograft mouse model [139]. LtxA was highly effective for all parameters measured including epidermal thickness, clinical psoriasis score, and lymphocyte infiltration. Ex vivo studies on PBMCs from psoriasis patients identified that LtxA preferentially targeted CD4^+^CD25^+^ T lymphocytes and CD14^+^CD16^+^ monocytes in these patients [139]. This subset of T cells [119,140] and monocytes [141] have previously been implicated in the progression of psoriasis. Therefore, LtxA may provide therapeutic benefits and resolution of psoriatic plaques with fewer, if any, adverse side effects.

### 4.3. Allergic Asthma

Asthma is an inflammatory disease of the airways that affects millions of people worldwide and can cause mortality. Asthma progresses due to infiltration of activated WBCs into the airways, and the release of inflammatory mediators from these cells that subsequently damage the bronchial epithelium [142]. Inflammation in asthma is dependent on the migration of WBCs into the airways. This migration is mediated by LFA-1, which has been shown to be overexpressed on immune cells from asthma patients [113,114]. Investigations into the therapeutic potential of LtxA for treatment of allergic asthma found that PBMCs from asthma patients have a unique population of CD4^−^CD11a^hi^ cells, the majority of which were CD14^+^ monocytes, that are preferentially targeted by LtxA [114]. In a mouse model for allergic asthma, LtxA caused resolution of disease, as demonstrated by a decrease in bronchoalveolar lavage fluid WBCs, a reduction in pulmonary inflammation and tissue remodeling, as well as a decrease in proinflammatory cytokines associated with asthma in lung tissue [114]. Thus, LFA-1 may serve as a biomarker in allergic asthma and the use of LtxA to treat this disease could be a useful therapeutic strategy.

### 4.4. HIV

Human immunodeficiency virus (HIV) infects CD4^+^ T cells, macrophages, and dendritic cells. HIV infection leads to progressive loss of CD4^+^ T cells and eventually, the loss of cell-mediated immunity, leading to the development of AIDS. LFA-1 and its ligand, ICAM-1, have been shown to be incorporated into the envelopes of budding HIV-1 virions from activated primary CD4^+^ T cell [116,117]. HIV-1 virions possessing ICAM-1 are more infectious due to the ability to bind LFA-1 on target cells [143,144]. These molecules play a crucial role in cell–cell transmission of HIV since they are components of the virological synapse [145,146]. The HIV envelope protein gp120 induced the activation of LFA-1 in naïve CD4^+^ T cells, making these cells more susceptible to HIV infection [65,115]. Virus p24-expressing CD4^+^ T cells isolated from PBMCs of HIV infected patients expressed higher surface levels of active LFA-1 compared to p24^−^ T cells from the same patients [65]. Ex vivo treatment of HIV-infected PBMCs with LtxA led to a significant reduction in viral DNA burden [65], suggesting that while HIV infection may activate CD4^+^ T cells, doing so makes them more susceptible to LtxA. Therefore, LtxA may be utilized as a therapeutic strategy to deplete HIV viral reservoirs in infected individuals who are managing the disease with antiretroviral therapy.

### 4.5. The Role of β_2_ Integrins in Other Autoimmune and Inflammatory Disorders

Alopecia areata is an autoimmune disorder characterized by damage to hair follicles and subsequent loss of hair [147]. Damage to hair follicles is cause by infiltration of activated, pro-inflammatory WBCs migrating to the follicles. Activated T cells, NK cells, eosinophils and mast cells have all been implicated in the progression of alopecia [118,119,120,121,122]. These immune cell subsets all express LFA-1 and other β_2_ integrins, thus, LtxA could be a potent and safe anti-inflammatory agent for the treatment of alopecia [148].

Systemic lupus erythematosus is an autoimmune disorder whereby a patient’s own immune cells become hyperactive and begin to attack healthy tissues, producing widespread inflammation [149]. Lupus is characterized by the immune system producing autoantibodies against itself [150]. Autoantibody complexes and immune cells can cause damage to many systems of the body, including joints, kidneys and blood cells. Treatments for lupus typically include immunosuppressive drugs, which can result in a higher frequency of opportunistic infections in these patients. T- and B-lymphocytes from patients with lupus were found to have increased expression of LFA-1 [123,124,125,126]. Additionally, PBMCs isolated from patients with lupus were found to have higher expression levels of Fas [151]. Therefore, LtxA may be a novel therapeutic option for lupus patients that is not immunosuppressive and leads to better health during treatment plans.

Multiple sclerosis is an autoimmune disorder affecting the central nervous system. Autoreactive immune cells destroy the myelin sheath surrounding nerve cells in the brain and spinal cord [152]. This disrupts communication from the brain to the rest of the body. There is no cure for multiple sclerosis and treatment is generally with immunosuppressive agents. LFA-1 was found to be highly expressed on immune cells in the peripheral blood and central nervous system of patients with multiple sclerosis [127,128]. In support of targeting integrins to treat multiple sclerosis, the United States Food & Drug Administration (FDA) approved Tysabri (Natalizumab), a monoclonal antibody that targets the cell adhesion molecule α_4_-integrin. Tysabri prevents the migration of inflammatory immune cells across the blood-brain barrier. Therefore, LtxA may be a beneficial therapeutic agent for the treatment and/or management of multiple sclerosis by targeting LFA-1 and removing these pro-inflammatory cells from circulation.

Inflammatory bowel disease (IBD) refers to chronic inflammatory disorders of the digestive tract. Ulcerative colitis and Crohn’s disease are the most common forms of IBD. Ulcerative colitis and Crohn’s disease are autoimmune diseases in which the body’s immune cells attack regions of the digestive tract. Ulcerative colitis manifests as ulcers in the lining of the colon and rectum, while Crohn’s disease can affect the small intestine, large intestine, and stomach [153,154,155]. IBD is typically treated with immunosuppressive agents. In patients with ulcerative colitis or Crohn’s disease, leukocytes in the intestinal tissues have been found to have enhanced LFA-1 expression, which could lead to an enhanced migration of autoreactive cells from peripheral blood to intestinal tissues [129,130,131,132]. Therefore, ulcerative colitis and Crohn’s disease may be treatable with LtxA and ongoing studies are investigating this possibility.

Dry eye disease is a common ocular disorder that affects 5–34% of the population [156]. Dry eye disease is believed to be an antigen-specific autoimmune disease associated with inflammation of the lacrimal gland and ocular surface [157,158]. Typically, this results in the inability to produce adequate tears, leading to dryness and eye pain. Although no causative antigen has been identified, the pathophysiology is believed to be caused by antigen-presenting cells priming naïve T cells into CD4^+^ T_H_1 and T_H_17 cells that migrate to the ocular surface [157,158,159,160]. In order for these autoreactive T cells to migrate to the ocular surface, they must have enhanced expression of the activated form of LFA-1 [133,161,162]. The FDA approved Xiidra (Lifitegrast), a small molecule inhibitor of LFA-1, for treatment of dry eye. Xiidra blocks LFA-1 interaction with its natural ligand, ICAM-1 [159,160,163,164], thus diminishing the migration of inflammatory T cells to the ocular surface. Due to the effectiveness of inhibiting LFA-1 and the enhanced expression of LFA-1 in dry eye, LtxA therapy may be beneficial to patients suffering from dry eye disease by eliminating these activated LFA-1-expressing cells.

LtxA may have significant therapeutic potential for the treatment of various autoimmune and inflammatory diseases. LtxA would provide a great benefit over many currently available therapeutics because it is not immunosuppressive and the agent selectively eliminates activated immune cells that contribute to the pathologies in the above-mentioned diseases. However, more investigations are necessary to provide data for proof-of-principle in other autoimmune disorders.

## 5. Summary and Conclusions

In summary, we described how LtxA initially interacts with target cell membranes in a cholesterol-dependent manner. LtxA is therefore able to interact with LFA-1 or other β_2_ integrins. LtxA/LFA-1 interaction triggers intracellular signaling pathways, which ultimately leads to cell death. The cell death mechanism varies by immune cell subset. We also discussed preliminary pre-clinical efficacy data supporting the development of LtxA as a novel biologic for the treatment of various hematological malignancies and immune-mediated diseases.

## Figures and Tables

**Table 1:** The role of β_2_ integrins in hematological malignancies and immune-mediated diseases.

Indication	Integrins	References
Leukemia	α_L_β_2_	[107,109,110]
Lymphoma	α_L_β_2_	[107,109,110]
Psoriasis	α_L_β_2_, α_M_β_2_, α_X_β_2_	[111,112]
Asthma	α_L_β_2_	[113,114]
HIV	α_L_β_2_	[65,115,116,117]
Alopecia Areata	α_L_β_2_,	[118,119,120,121,122]
Systemic Lupus Erythematosus	α_L_β_2_, α_M_β_2_	[123,124,125,126]
Multiple Sclerosis	β_2_	[127,128]
Crohn’s Disease	α_L_β_2_, α_M_β_2_	[129,130,131,132]
Ulcerative Colitis	α_L_β_2_, α_M_β_2_	[129,130,131,132]
Dry Eye	α_L_β_2_,	[133]
